# Profiles of women’s adjustment after cancer based on sexual and psychosocial wellbeing: results of a cluster analysis

**DOI:** 10.1186/s12885-022-10093-6

**Published:** 2022-09-21

**Authors:** Elizabeth K. Arthur, Usha Menon, Jennifer Barsky Reese, Kristine Browning, Janine Overcash, Karen Rose, Celia E. Wills

**Affiliations:** 1 The Ohio State University, College of Nursing, 1585 Neil Ave, Columbus, OH 43210 USA; 2 University of South Florida, College of Nursing, 12901 Bruce B. Downs, MDN 22, Tampa, FL 33612-4742 USA; 3 Fox Chase Cancer Center, Cancer Prevention and Control Program, 333 Cottman Ave, Philadelphia, PA 19093 USA

**Keywords:** Sexual function, Sexual communication, cancer, Oncology, Sexual distress, Self-efficacy

## Abstract

**Background:**

Sexual wellbeing is a critical yet often overlooked aspect of overall wellbeing for women across cancer diagnoses.

**Objective:**

We identified profiles of women cancer survivors by sexual and psychosocial outcomes and compared groups for differences in relevant outcomes and individual characteristics.

**Methods:**

Partnered women treated for cancer (*n* = 226; M age = 51.1 (12.6); 54% breast cancer; 86% White) completed a cross-sectional survey assessing sexual and psychosocial wellbeing. K-means cluster analysis modeled subgroups (clusters) with similar response patterns on measures of sexual wellbeing (sexual function, distress, sexual communication, and self-efficacy for communication), psychosocial wellbeing (quality of life (QOL), anxiety and depressive symptoms), and time since treatment. ANOVAs with Tukey post-hoc analyses and chi-square analyses tested cluster mean differences.

**Results:**

Three distinct clusters of women differed by levels of adjustment in sexual and psychosocial wellbeing: higher-adjustment (32.7%), intermediate (37.6%), and lower-adjustment (29.6%). Significant differences among the clusters were found for all outcomes, with largest effect sizes for sexual distress (η^2^_p_ = 0.66), sexual communication (η^2^_p_ = 0.51), sexual satisfaction (η^2^_p_ = 0.44), and anxiety and self-efficacy for communication (η^2^_p_ = 0.32). The intermediate adjustment group was characterized by lower adjustment on measures of sexual and relationship function, and better adjustment on measures of QOL and mood.

**Conclusions:**

Findings suggest that for women cancer survivors, measures of sexual and psychosocial wellbeing can model distinct profiles to inform targeted interventions to meet women’s needs. Evidence-based targeted interventions could lead to better sexual function, and ultimately to better QOL and overall wellbeing.

**Implications for practice:**

A stepped intervention approach to sexual health care for women with cancer, where content and format depend on degree of sexual and psychosocial adjustment after cancer, may be most appropriate. Interdisciplinary teams may address sexual, emotional, and relationship functioning.

There are approximately 8.8 million women with a history of invasive cancer in the United States [1], many of whom experience long term sexual sequelae of their treatment [2, 3]. Sexual wellbeing is an important part of quality of life for women across different cancers or treatment types [4], ages [5], and menopausal statuses [6]. Cancer treatment may cause physical, emotional, and relational changes that negatively affect sexual function and intimacy in relationships. Despite evidence that women want these issues addressed [7, 8], few oncology clinicians feel they have the time, expertise, comfort level, or referral resources to address women’s sexual wellbeing [9, 10]. Thus, women’s sexual wellbeing after cancer treatment is largely unaddressed [11].

Sexual wellbeing is a multidimensional concept [12], comprised of physical, emotional, and interpersonal components as evidenced by the relationship of sexual function with mood, relationship quality, self-efficacy, and communication [13, 14]. While prior research has shown that women’s sexual and relationship function, mood, and QOL are inter-related, studies have not specifically examined whether women could be grouped, or profiled, according to their responses on these outcome measures. Identifying profiles based on scores on outcomes of sexual, relationship, and psychosocial well-being could assist women and/or their clinicians in the selection of existing interventions, or inform the development of interventions to best address women’s sexual health needs to improve their overall well-being. For instance, it is possible that some women could experience sexual dysfunction and distress concurrently with distressed mood and relationship quality, whereas others may experience mild concerns, with limited or no concomitant effects seen for their mood or relationship quality. This individual variation in experiences may call for different approaches to education, support and treatment. Therefore, the goal of the present study was to explore sexual and psychosocial wellbeing of adult women treated for cancer, and to characterize subgroups (profiles) of women via cluster analysis based on standardized measures of sexual and psychosocial outcomes.

## Methods

This manuscript describes a secondary analysis of cross-sectional data collected from 226 women with a cancer history who participated in an instrument evaluation study [14]. The goal of the original study was to assess the psychometric performance of the newly developed Self-Efficacy to Communicate about Sex and Intimacy (SECSI) scale which is intended to assess a woman’s confidence in her ability to communicate with her intimate partner about sex and intimacy. The university institutional review board approved the study (IRB #2016B0341). Adult women treated for any type of cancer who had a male partner were eligible. Women were recruited through local media (flyers, newsletters, oncology clinic postings), and ResearchMatch®, a NIH-sponsored national database of health research volunteers. Interested women were sent a link to complete informed consent, and to respond to standardized measures and questions about sexual behaviors, sociodemographic, and cancer characteristics via a one-time online survey.

### Measures

Sexual Function. The Female Sexual Function Index (FSFI) is a widely used 19-item self-report scale, with higher scores indicating better sexual functioning [15] that has significant research supporting its use in women with cancer [16]. The FSFI had high internal consistency reliability in the present sample (Cronbach’s alpha = 0.97 for the total scale, and 0.85 - 0.98 for the subscales).

Sexual Distress. The Female Sexual Distress Scale Revised (FSDS-R) is a 13-item scale assessing sexually-related personal distress in women [17] that has been used successfully in women with cancer [18, 19]. The FSDS had high internal consistency reliability in this sample (Cronbach’s alpha = 0.96).

Sexual Behaviors. This 10-item questionnaire covered sexual behaviors (sex or intimate act) over the last 4 weeks [14]. They were asked if they had interest in or engaged in sexual activity (yes/no); if they or their partner had avoided or declined sexual advances, and if their partner had any limitations to perform sexual activity (yes/no)’ frequency of kissing and intercourse (frequency scale from none to more than once per day), and which partner initiated these activities most of the time; and to rate their sex life currently from ‘could not be better’ to ‘could not be worse’.

Relationship Satisfaction. Relationship satisfaction was measured using the 7-item abbreviated version of the Dyadic Adjustment Scale (DAS-7), which has been shown to correlate strongly with the original measure [20]. Higher scores indicate better relationship quality and satisfaction. The DAS-7 had good internal consistency reliability in the present sample (Cronbach’s alpha = 0.83).

Sexual Communication. The 13-item Dyadic Sexual Communication Scale (DSCS) has demonstrated good internal consistency reliability (Cronbach’s alpha = 0.81), test-retest reliability (*r* = 0.89), and discriminant validity between people with and without sexual problems (*p* < .01) [21, 22]. Higher scores indicate better perceived quality of communication. The DSCS had excellent internal consistency reliability in the present sample (Cronbach’s alpha = 0.90).

Self-Efficacy for Sexual Communication. The Self-Efficacy to Communicate about Sex and Intimacy (SECSI) scale is a 10-item scale assessing the extent of confidence to communicate with a romantic partner about changes in sex and intimacy after cancer treatment, with higher sum scores indicating greater self-efficacy [14]. The SECSI scale had high internal consistency reliability (Cronbach’s alpha = 0.94) and good test-retest reliability (*r* = 0.82) in the reported study.

Health-Related Quality of Life. Health-related quality of life was measured by the widely used Functional Assessment of Cancer Therapy–General (FACT-G) instrument [23] which contains 27 items assessing four domains of wellbeing: physical, social/family, emotional, and functional. Higher scores signify higher perceived quality of life. The FACT-G had very good to excellent internal consistency reliability in the present sample (Cronbach’s alpha = 0.93 for the total scale, and 0.84 – 0.88 for subscales).

Anxiety and Depression. Anxiety and depression symptoms were measured using the Generalized Anxiety Disorder (GAD-7) [24] scale and Patient Health Questionnaire (PHQ-8) scale [25]. Higher scores indicate greater symptom burden. Both scales had very good to excellent internal consistency reliability in the present sample (Cronbach’s alpha = 0.91 for the GAD-7 and 0.87 for the PHQ-8).

### Data analysis

Degree and patterns of data missingness was evaluated by variable. Standardized scales with 5% missing data or less were assumed to be missing at random, and therefore missing values were imputed for analysis. We used multiple imputation methods to model missing data for GAD-7, PHQ-8, FSDS, SECSI, DAS-7, and DSCS. It appeared that women with missing FACT-G data (7.5-15.5% missing data by subscale) and FSFI data (25.7%) appeared to be missing not at random and therefore cases were retained in models but missing data was not imputed.

K-means cluster analysis was used to model subgroups with similar response patterns on measures of sexual wellbeing (sexual function, distress, sexual communication, and communication self-efficacy), psychosocial wellbeing (quality of life (QOL), anxiety and depressive symptoms), and time since treatment. Main study variables initially entered were subsequently reduced until there were several technically viable cluster solutions. *K*-means cluster analysis generated several technically viable cluster solutions that included two, three, and four derived clusters. The three-cluster model is presented in Table 2. The three-cluster solution best-balanced technical, conceptual, and practical considerations, incorporating the anticipated clinical and research uses of the results.

Once the clusters were identified, we tested for significant differences in cluster means. For interval and ratio variables, we first evaluated group differences with one-way analysis of variance (ANOVA) followed by *post-hoc* Tukey analysis for control of Type I error. Then partial eta-squared (*η*^*2*^_*p*_) values were computed to examine the relative effect sizes of mean scores differences between clusters on modeled variables. For categorical variables, chi-square tests of proportions (χ^2^) were used to examine group differences. Discriminant function analysis was performed to validate cluster analysis results.

## Results

The mean age in this study was 51 years old (SD 12.6). Women were together with their partner an average of 20 years (SD 13.8). Most women (86.3) identified as white and highly educated with 53.5% having experienced breast cancer (Table 1). Sixty-eight percent of participants participated in sexual or intimate activity in the last 4 weeks.

**Table 1:** Demographic Characteristics of Total Sample and Clusters (*n =* 226)

Measure (Range)	Total Sample (***N =*** 226)	Cluster 1 (***n =*** 74) Higher Adjustment	Cluster 2 (***n =*** 85) Intermediate Adjustment	Cluster 3 (***n =*** 67) Lower Adjustment
	**Mean ± SD**	**Mean ± SD**	**Mean ± SD**	**Mean ± SD**
**Age**^**a**^ **(21 – 86)**	51.1 ± 12.6	52.9 ± 13.8	52.1 ± 12.4	47.8 ± 11.0
**Years with Partner**^**b**^ **(1 - 65)**	20.0 ± 13.8	19.3 ± 14.8	22.9 ± 14.2	17.2 ± 11.6
**Years Since Last Treatment**^**c**^ **(0 – 36)**	4.3 ± 5.7	5.0 ± 6.6	4.3 ± 5.2	3.6 ± 5.3
**FSFI**	16.4 ± 10.0	24.3 ± 9.6	14.7 ± 8.8	11.1 ± 7.0
**FSDS**	19.3 ± 13.6	8.8 ± 7.9	15.6 ± 7.9	36.0 ± 8.1
**DAS-7**	24.0 ± 5.3	26.7 ± 3.9	22.9 ± 5.0	22.3 ± 5.9
**DSCS**	54.4 ± 13.2	67.8 ± 6.1	47.4 ± 8.1	48.6 ± 12.9
**SECSI**	17.8 ± 6.9	23.5 ± 5.2	16.1 ± 5.0	14.3 ± 6.8
**FACT-G Physical**	22.3 ± 5.5	24.0 ± 5.2	23.1 ± 4.9	19.3 ± 5.6
**FACT-G Social/Family**	20.0 ± 5.7	23.2 ± 4.3	20.4 ± 5.0	16.0 ± 5.4
**FACT-G Emotional**	18.5 ± 4.5	20.5 ± 2.9	19.1 ± 3.7	15.5 ± 5.4
**FACT-G Functional**	19.8 ± 5.9	21.8 ± 5.5	21.0 ± 5.0	16.0 ± 5.8
**GAD-7**	5.1 ± 4.8	2.6 ± 3.1	4.0 ± 4.0	9.0 ± 4.7
**PHQ-8**	5.5 ± 5.1	3.4 ± 3.8	4.2 ± 3.6	9.5 ± 5.1
	**(N) Percent**	**(N) Percent**	**(N) Percent**	**(N) Percent**
**Race/Ethnicity**^**d**^				
White	(195) 86.3	(63) 86.3	(73) 88.0	(59) 93.7
Other race, ethnicity or origin	(24) 10.6	(10) 13.7	(10) 12.0	(4) 6.3
**Education Level**^**e**^				
Some college, vocational, or Associates Degree	(52) 23.0	(17) 24.6	(16) 21.1	(19) 31.7
Bachelor’s Degree	(80) 35.4	(23) 33.3	(30) 39.5	(27) 45.0
Master’s Degree	(51) 22.6	(21) 30.4	(21) 27.6	(9) 15.0
Post Master’s or Professional Degree	(22) 9.7	(8) 11.6	(9) 11.8	(5) 8.3
**Household Income**^**f**^				
$10,000 - $49,999	(37) 16.4	(14) 21.2	(10) 13.5	(13) 22.4
$50,000 - $99,999	(79) 35.0	(25) 37.9	(27) 36.5	(27) 46.6
$100,000 - $149,999	(48) 21.2	(18) 27.3	(16) 21.6	(14) 24.1
$150,000 or more	(34) 15.0	(9) 13.6	(21) 28.4	(4) 6.9
**Employment Status**^**g**^				
Not employed	(21) 9.3	(6) 8.5	(7) 9.3	(8) 12.5
Part-time	(40) 17.7	(12) 16.9	(18) 24.0	(10) 15.6
Full-time	(101) 44.7	(34) 47.9	(35) 46.7	(32) 50.0
Retired	(36) 15.9	(16) 22.5	(14) 18.7	(6) 9.4
Other	(12) 5.3	(3) 4.2	(1) 1.3	(8) 12.5
**Cancer Type**^**d**^				
Breast	(121) 53.5	(36) 50.0	(48) 56.5	(37) 55.2
Thyroid	(21) 9.3	(4) 5.6	(11) 12.9	(6) 9.0
Gynecologic	(30) 13.2	(12) 16.7	(7) 8.2	(11) 16.4
Melanoma	(13) 5.8	(5) 6.9	(6) 7.1	(2) 3.0
Colon	(8) 3.5	(4) 5.6	(1) 1.2	(3) 4.5
Other	(39) 13.8	(11) 15.3	(12) 14.1	(8) 11.9
**Sexually Active**^**h**^	(153) 68.9	(62) 83.8	(48) 57.8	(43) 66.2

Missing data, n (%): a = 5 (2.2); b = 9 (4.0); c = 2 (0.9); d = 7 (3.1); e = 21 (9.3); f = 28 (12.4); g = 16 (7.1), h = 4 (1.8)

*K*-means cluster analysis generated several technically viable cluster solutions that included two, three, and four derived clusters of women’s sexual and psychosocial wellbeing. The three-cluster model was the best representation of conceptual and practical considerations (Table 1). Overall, cluster 1 (32.7% of the sample) is characterized by better scores on sexual and psychosocial outcome measures, cluster 3 (29.6%) is characterized by worse scores on these measures, and cluster 2 (37.6%) is characterized by scores intermediate to these two groups on either extreme. The mean distance of cases in relation to the cluster centroid is an indicator of within-group homogeneity. The mean distances (with standard deviation) that were computed for the present sample to describe homogeneity within clusters are 16.7 ± 5.9 for cluster 1, 17.4 ± 4.8 for cluster 2, and 20.9 ± 6.2 for cluster 3.

There were statistically significant mean score differences between the three clusters for all variables except for time since treatment and time since diagnosis (Table 2). Effect sizes for statistically significant differences in variables between clusters range from small partial eta-squared (η^2^_p_) effects for age and years with partner (.03), medium η^2^_p_ for physical QOL and relationship satisfaction (.13), to large η^2^_p_ effects for the remainder of the modeled variables (.17 to .66). Most variables had a large effect size (*Ƞ*^*2*^ > 0.14): FACT-G Social (0.26), Emotional (0.20), Functional (0.17), and Total (0.26) scores; GAD-7 (0.32); PHQ-8 (0.28); FSFI Desire (0.23), Arousal (0.26), Lubrication (0.17), Orgasm (0.19), Satisfaction (0.44), Pain (0.17) and Total (0.29) scores; FSDS (0.66); SECSI (0.32); and DSCS (0.51). The largest effect size for difference between cluster means was for sexual distress with η^2^_p_ of 0.66. The clusters of women also differed significantly on several sexual behaviors over the last 4 weeks (*p*-values.05-.01): interest in sexual activity (yes/no), engagement in sexual activity (yes/no), whether they avoided or declined sexual advances from their partner (yes/no), frequency of kissing, frequency of intercourse or equivalent activity, and ratings of their sex life.

**Table 2:** Differences in Variables by Cluster Membership

Variable	***df***	***F***	***Ƞ***^***2***^	***p***
Years since diagnosis	2	0.076	0.001	0.927
Year since treatment	2	1.032	0.009	0.358
FACT-G Physical Wellbeing^b,c^	2	15.623	0.125	0.000
FACT-G Social Wellbeing^a,b,c^	2	36.567	0.262	0.000
FACT-G Emotional Wellbeing^b,c^	2	24.572	0.204	0.000
FACT-G Functional Wellbeing^b,c^	2	19.731	0.173	0.000
FACT-G Total^b,c^	2	30.792	0.263	0.000
GAD-7^b,c^	2	51.532	0.319	0.000
PHQ-8 ^b,c^	2	43.350	0.284	0.000
FSFI Desire Subscale^a,c^	2	28.197	0.227	0.000
FSFI Arousal Subscale^a,c^	2	29.204	0.255	0.000
FSFI Lubrication Subscale^a,c^	2	17.041	0.169	0.000
FSFI Orgasm Subscale^a,c^	2	19.470	0.188	0.000
FSFI Satisfaction Subscale^a,b,c^	2	66.476	0.443	0.000
FSFI Pain Subscale^a,c^	2	17.059	0.170	0.000
FSFI Total^a,c^	2	32.829	0.285	0.000
FSDS^a,b,c^	2	208.750	0.663	0.000
SECSI^a,c^	2	34.954	0.316	0.000
DAS-7^a,c^	2	16.277	0.133	0.000
DSCS^a,c^	2	108.382	0.507	0.000
Age^c^	2	3.198	0.029	0.043
Years with partner^b^	2	3.268	0.030	0.040

Legend: ^a^, significant difference between clusters one and two; ^b^, significant difference between clusters two and three; ^c^, significant difference between clusters one and three; *p* < 0.05. Eta-squared values in theory can range from 0 (no difference) to 1 (maximally different). Cohen’s conventions for interpretation of η^2^ values suggest that effect sizes of about .01 are small, .06 to .14 are medium, and above .14 are large [26]

Caption: This table displays results from the three cluster ANOVA with Tukey post-hoc analyses for differences in variable means

**Fig. 1:**
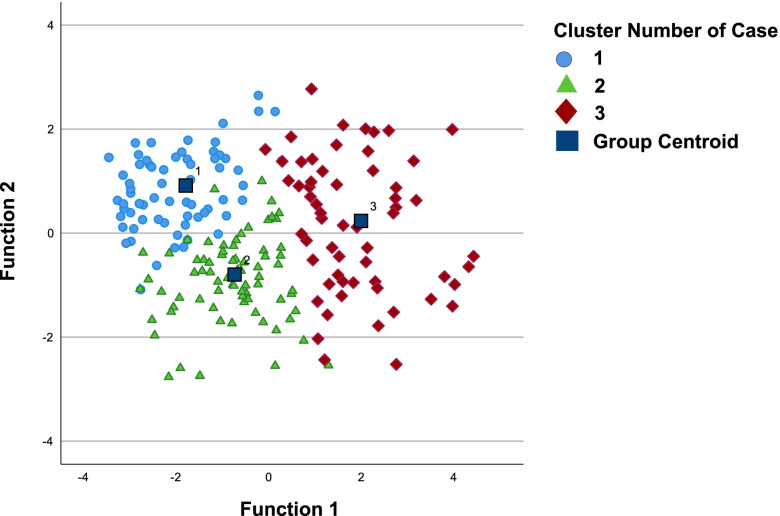
Case Distribution by Cluster. Legend: Canonical discriminant functions are plotted with cases assigned to clusters (blue circles = cluster 1, green triangles = cluster 2, red diamonds = cluster 3). Cluster centroids (dark blue squares) are included to illustrate cluster dispersion and homogeneity

In a stepwise discriminant function analysis (for a review of clustering methods and validation techniques, see Aldenderfer and Blashfield [27], the model retained FSDS, DSCS, and GAD-7, and removed the other variables. Canonical discriminant functions are plotted with cases assigned to clusters (Fig. 1). Cluster centroids are included to illustrate cluster distribution and homogeneity. Classification results showed that the predictive model would correctly assign cases to cluster 1 with an accuracy of 86.8%, cluster 2 with an accuracy of 97.3%, and cluster 3 with an accuracy of 94.9%.

## Discussion

Whereas most prior quantitative studies have examined correlations between and predictors of sexual wellbeing, mood and QOL [28, 29], this study is unique in examining distinct subgroups of women who may share certain sexual and psychosocial factors. The cluster analytic approach used in the present study allows us to extend previous findings by characterizing subgroups (profiles) of women who differ in their sexual and psychosocial wellbeing and risk factors.

Overall, study findings are consistent with prior research in women with cancer showing that women reporting higher versus lower sexual well-being tend to differ in their physical and psychological health outcomes [30]. For instance, prior qualitative studies have demonstrated that women report a range of experiences in response to cancer survivorship, from significant negative consequences of treatment on women’s sexual function and relationship to growing closer together with a partner and finding new ways for experiencing intimacy [31, 32]. Our findings are also consistent with results of prior quantitative studies demonstrating that mood, sexual wellbeing and relationship quality are generally correlated for women after cancer [33, 34]. In this vein, a number of studies have found that cancer survivors who have better sexual communication are more responsive to each other’s needs, may engage in more effective coping, and experience less distress, better sexual functioning, and report better relationship satisfaction [29, 35, 36] than women with poor sexual communication.

A key finding that extends previous research was that three distinct subgroups of women treated for cancer emerged that differed in their reports of sexual and psychosocial wellbeing. Slightly over one-third of the sample fell into cluster 1, the higher-adjustment group, which was characterized by better sexual adjustment and relationship adjustment, mood, and quality of life. Women in this cluster were also distinguished by greater self-efficacy, or confidence, in communicating with their partners about changes in sex and intimacy after cancer treatment. In sum, they were doing very well in a range of domains of sexual and overall health. The largest group, however, was cluster 2, the intermediate adjustment group, which was characterized by lower adjustment on measures of sexual and relationship function, but better adjustment on measures of QOL and mood. The findings for this group are intriguing because they indicate that the lower adjustment in the sexual wellbeing outcomes for women in this group seem independent of their mood or QOL. Women in this group may be engaging in coping skills that prevent their sexual problems from impairing their mood or QOL. Another possibility is thatthey may have had high premorbid mood, which could have acted as a buffer against the impact of sexual problems on their overall wellbeing. Finally, women in cluster 3, the lower-adjustment group, reported worse sexual wellbeing, worse mood, and worse quality of life compared to the other two subgroups of women, suggesting that women in this group may be struggling with many aspects of recovery and survivorship extending beyond their sexual lives. This is the group that may have the least amount of resources, whether in terms of their mood or relationship, to call upon to buffer against their sexual problems, leading to worse overall outcomes, although this is conjecture.

One noteworthy finding was that sexual distress had the largest effect size difference between groups, signifying that it was the measure that best distinguished the clusters of women. The fact that this measure tracked most closely with the levels of adjustment represented by the clusters may be partly explained by the nature of sexual distress as capturing a higher-level appraisal of the sexual problems women are experiencing [37, 38]. For instance, whereas some women may experience low desire and feel distressed as a result, others may experience the same low desire without singificant distress. Indeed, distress constitutes an important component of sexual disorders and one that clinical guidelines in oncology recommend should be assessed when screening for sexual function problems. Our findings are in line with these clinical recommendations and suggest that sexual distress may serve as a useful barometer for women’s overall cancer-related sexual adjustment [39].

When differences between clusters in demographic characteristics were examined, there appeared to be some patterns that may be helpful to describe the groups. Both age (cluster 1 members more likely to be older than cluster 3 members, *p* = 0.04) and years in relationship with their partner (cluster 2 members more likely to be in a longer relationship than cluster 3 members, *p* = 0.04) differed significantly between groups. Other differences in demographics by cluster were not significant, but trends may bear examining, such as the larger proportion of women in cluster 3 in the lower education and income categories. Further research is warranted to understand how social and economic factors influence psychological and sexual adjustment after cancer.

Results of this secondary data analysis should be interpreted in light of the study’s strengths and limitations. The findings are generalizable to a relatively broad cancer survivor population in the U.S., as participants represented a wide age range and types of cancer. This is a strength because most prior studies have tended to focus on survivors of breast and gynecologic cancer [2, 40]. Yet, individuals diagnosed with non-reproductive cancers can rate sex as equally important as those with cancers affecting the reproductive organs (e.g., breast and gynecologic cancers) [29], supporting the premise that sexual wellbeing should be studied in women across a range of cancer diagnoses. A limitation of the sexual function (FSFI) scoring is that women who are not sexually active will have low scores (e.g., items about arousal, orgasm, or pain scored as ‘0’), though they may be sexually inactive for many reasons, including limitations of their partner to participate in sexual activity. Future studies should consider ways of addressing this issue, whether by limiting enrollment based on sexual activity status, using measures that can assess sexual function irrespective of sexual activity status, or including additional questions about reasons for sexual inactivity and potentially considering these responses in analyses.

Although we took into account both conceptual and practical considerations when selecting the three-cluster analysis model for interpretation, alternative cluster solutions and interpretations may also be viable. There are multiple potential guidelines suggesting sample size for cluster analysis, and our full sample may have been underpowered. The sample size was not such that we could do subgroup analyses and this study should be replicated in a larger sample. In addition, because the original study was a cross-sectional survey of women with widely variable time since treatment, it is not possible to determine whether women’s characteristics, and thus the differences between clusters, represent pre-cancer personal and relationship characteristics or effects of cancer treatment. All participants were partnered, so inferences should be limited to partnered survivors. In addition to replication of findings in larger samples, we also suggest replication studies in samples with more socioeconomic and racial/ethnic diversity. Further research is needed to design and test the efficacy, effectiveness, and costs of tailored interventions based on level or degree of need. While many studies have focused on sexual health and fertility in younger cancer survivors, the largest – and growing – age group of cancer survivors are older adults. As fatigue, diabetes, and poorer self-rated health are found to be associated with decline in sexual wellbeing for older adults, assessing and intervening for sexual wellbeing in this patient population is also warranted [41]. Finally, we did not examine partner factors such as sexual function or mood, which could have a bearing on women’s sexual adjustment and should be examined in future studies.

### Clinical implications

These results have important clinical implications. Findings suggest that many women report positive psychosocial and sexual adjustment after cancer, which may come as welcome news to clinicians. For instance, clinicians may wish to incorporate the message that many women can still enjoy satisfying relationships and wellbeing even in the face of sexual difficulties with their patients, who may find this encouraging news. In addition, findings suggest that a stepped approach to sexual health care for women with cancer may be most appropriate and that such an approach could include different interventions, both in terms of content and format (e.g., duration, intensity), depending on the profile of adjustment. For example, whereas all women after cancer should receive assessment of cancer-related sexual concerns and education on effects of treatment on sexual function [42], a relatively small number of women will likely require intensive and complex sexual health treatment or sex and relationship therapy. Specifically, results of this study suggest that particular attention could be paid to efforts at assessing and managing sexual issues for women in the lower adjustment group, given how wide-ranging the impact of their cancer seems to be on various facets of their well-being. Clinicians may find it useful to consider whether a patient’s sexual difficulties are co-occurring with difficulties in mood and relationship quality, as this could suggest that an interdisciplinary approach addressing not only the patient’s sexual concerns, but also aspects of their emotional and relationship functioning could be beneficial. By contrast, we suspect that the women experiencing sexual difficulties but who report largely positive adjustment otherwise, could potentially benefit from a focused sexual wellbeing intervention. More work needs to be done to examine the content and format of interventions that would best meet the needs of each of these groups.

## Conclusion

Sexual difficulties are common and distressing after treatments for a range of cancers. While such difficulties are widely prevalent, the results of this study suggest they can be found both alongside and independent of other psychosocial difficulties, indicating a range of experiences for women. Examining profiles of response across a range of measures of wellbeing may help determine paths forward to intervention and ultimately enhance quality of life for women after cancer.

## Data Availability

The datasets generated during and/or analysed during the current study are available from the corresponding author on reasonable request.
